# The impact of the acute surgical assessment unit on the management of acute appendicitis: a single-centre review

**DOI:** 10.1007/s11845-021-02706-z

**Published:** 2021-07-11

**Authors:** Enda Hannan, Sherif El-Masry

**Affiliations:** Department of Surgery, Our Lady of Lourdes Hospital Drogheda, 109 Howth Road, Howth, Dublin 13, Ireland

**Keywords:** Acute appendicitis, Acute care surgery, Acute surgical assessment unit, Acute surgical unit, ASAU, ASU, Emergency department, Emergency general surgery, Laparoscopic appendicectomy

## Abstract

**Background:**

Acute surgical assessment units (ASAUs) aim to optimise management of surgical patients compared to the traditional ‘on-call’ emergency department (ED) system. Acute appendicitis (AA) is the most common acute surgical condition requiring emergency surgery.

**Aim:**

We set out to assess if the ASAU improved care provided to patients with AA compared to those managed through the ED.

**Methods:**

Patients admitted via the ED with AA in the 6 months prior to opening the ASAU were compared to those admitted via the ASAU in the first six months following its implementation. Relevant data was collected on key performance indicators from their charts.

**Results:**

In the ASAU cohort, the mean time to be seen was one hour less than the ED cohort (21 min vs 74 min). The mean time to surgery was also 8.8 h shorter. Most patients in the ASAU group (78.6%) underwent surgery during the day, compared to 40.3% of ED patients. The ASAU patients also had a lower postoperative complication rate (0.9% vs 3.9%), as well as a lower negative appendicectomy rate (14.2% vs 18.6%) and lower conversion-to-open surgery rate. Greater consultant supervision and presence was observed.

**Conclusions:**

The ASAU has resulted in better outcomes for patients with AA than those admitted via ED. More operations were performed in safer daytime hours with greater consultant presence, allowing for improved senior support for trainee surgeons. Our study supports the role of the ASAU in improving the quality and efficiency of emergency general surgery.

## Introduction

Emergency general surgery (EGS) represents more than 50% of the surgical workload and 80% of surgical deaths [1, 2]. The traditional means of managing EGS patients is the ‘on call’ system, whereby patients are assessed in the emergency department (ED) by a surgical team providing 24-h emergency cover. This is while also being expected to attend to elective duties such as theatre lists, ward rounds, outpatient clinics, surgical consults and administrative work. With such a heavy burden of tasks and without dedicated time on call, delays in care for acute surgical patients are unavoidable [1, 2]. With this comes negative outcomes for both patient safety and surgeon wellbeing [3]. The reasons for suboptimal care are multifactorial and may be attributed to lack of availability of on call staff, inconsistent consultant supervision and limited access radiology and operating theatre slots [1]. The traditional defence for this model of care has been that, by its very nature, emergency work is unpredictable. However, it has been shown that the emergency surgery workload is largely predictable, and thus it is possible to tailor services to account for this [4]. The separation of emergency and elective general surgery care has been widely advocated for as a critical means of improving the quality and efficiency of EGS [1, 5].

The acute surgical assessment unit (ASAU) represents a new model of care for the EGS workload [1–4]. The concept of the ASAU is to provide a dedicated area in the hospital separate to the ED where acute surgical patients can be assessed [6]. This is covered by an on-site consultant-led surgical team that is free from elective duties and solely responsible for the provision of emergency care with protected access to diagnostics and a dedicated emergency operating theatre [6]. The purpose of the ASAU is to provide patients with timely access to assessment, investigation and treatment by senior decision makers, thus improving patient care and clinical outcomes [1, 7]. The first formal ASAU was introduced in Australia and has subsequently been adapted in healthcare services worldwide [6]. In 2013, the National Clinical Programme in Surgery (NCPS) published the Acute Surgery Model of Care which introduced the ASAU model to Ireland, with six such units now operational across the country [6]. While studies on the impact of the ASAU on EGS have largely been favourable, those available are largely from Australasia, and there have been conflicting reports regarding certain KPIs such as time to theatre and the proportion of procedures being performed outside of normal working hours [1]. As the ASAU remains a relatively new venture in Ireland, literature on its success and shortcomings remains limited [6, 8–10].

Acute appendicitis (AA) is the one of the most common general surgical emergency presentations, with an incidence of around 100 cases per 100,000 person years [11]. While there has been some interest in conservative management, the laparoscopic appendicectomy remains the most widely accepted standard of care, and is the most commonly performed emergency general surgical procedure worldwide [9]. For this reason, AA has been used as a key performance indicator (KPI) condition to assess performance in the EGS setting [12].

## Aim

This study aimed to investigate the impact of the implementation of the ASAU model of care on the management of appendicitis in our hospital by comparing KPIs and clinical outcomes to that of the traditional ‘on call’ ED model of care that was in place prior to the opening of the ASAU. To our knowledge, this is the first study that specifically looks at the impact of implementation of the ASAU in an Irish hospital on outcomes in the management of AA.

## Methods

### Study design

A retrospective audit was performed involving patients who were admitted with a diagnosis of AA and subsequently managed by appendicectomy across two separate six month periods. These were the 6 months prior to the opening of the ASAU during which patients were managed by means of the traditional ‘on call’ model following presentation to ED and the first 6 months following implementation of the ASAU. Patients eligible for inclusion were over the age of 16 years, admitted with a diagnosis of AA and underwent an appendicectomy during their index admission. Patients admitted for an elective or interval appendicectomy, those with incomplete data and those that had appendicitis that was managed conservatively were excluded from the study.

### Structure of the ASAU

The ASAU was opened in October 2015 in our centre. It is a six-bed unit that is open from 8 am to 8 pm 7 days a week. It is covered by the rostered consultant on call, with a dedicated on-site surgical registrar and senior house officer that are free from all other clinical commitments providing SAU cover. It is also staffed by three surgical nurses trained in phlebotomy and cannulation and a healthcare assistant. Patients are referred to the ASAU by nursing triage in ED, and the referrals are screened by the ASAU registrar. A small number of dedicated radiology slots for both ultrasonography (US) and computed tomography (CT) are provided daily for SAU patients. The acceptance criteria for the ASAU includes all patients with a variety of common acute general surgical conditions, including appendicitis. Patients that need resuscitation room level care or that are haemodynamically unstable are excluded from ASAU management. A dedicated emergency surgery theatre existed both before and after implementation of the ASAU, which is shared between general surgery, orthopaedics and urology. The rostered consultant on call was on-site from 8 am to 5 pm and performed a dedicated evening ward round in the ASAU.

### Data collection

Patients diagnosed with AA during the two study periods were identified using hospital inpatient enquiry (HIPE) data, the ASAU referral logbook and operating theatre logbooks. The performance of an emergency appendicectomy on patients diagnosed with AA was confirmed by review of the operating theatre logbooks. Following this, an in-depth review of medical records, operation notes, discharge summaries, radiology reports and histopathology reports was performed for those that met the inclusion criteria. Data was collected on patient demographics and the following outcomes:

- Time be seen by the surgical team from the time of referral
- Time from hospital presentation to the operating theatre
- The utilisation of preoperative imaging to aid in diagnosis of AA
- The time of day surgery was performed
- Level of consultant supervision in surgery
- Conversion of laparoscopic surgery to open surgery
- The presence of perforated appendicitis
- Inpatient length of stay
- Post-operative complications
- Thirty-day readmission, reoperation and mortality rates
- The negative appendicectomy rate

### Statistical analysis

Statistical analysis was performed using the software package SPSS (SPSS Inc, Chicago, IL). For continuous data, the independent samples *t*-test was used. For non-normally distributed data, the Mann–Whitney *U* test was used. A *p* value of less than 0.05 was considered statistically significant.

### Ethical approval

The study was registered with the hospital audit committee. All patient data was anonymised for the purpose of this study. No identifying information was retained by the authors or included in this article.

## Results

### Patient demographics (Table 1)

**Table 1:** Patient demographics

Overall demographics	
Number of patients	208
Male-to-female ratio	55:45
Mean age in years	28 (range 16–57)
ASA grade	− ASA I: 73.6% (n = 153) − ASA II: 21.6% (n = 45) − ASA III: 4.8% (n = 10)
**ASAU group demographics**	
Number of patients	106
Male-to-female ratio	57: 43
Mean age in years	29 (16–57)
ASA grade	− ASA I: 72.6% (*n* = 77) − ASA II: 21.7% (*n* = 23) − ASA III: 5.7% (*n* = 6)
**ED group demographics**	
Number of patients	102
Male-to-female ratio	53:47
Mean age in years	27 (range 16–53)
ASA grade	− ASA I: 74.5% (*n* = 76) − ASA II: 21.6% (*n* = 22) − ASA III: 3.9% (*n* = 4)

In the 6 months prior to opening the ASAU, 102 patients were admitted through the ED with a diagnosis of AA which was managed by appendicectomy (ED group). The mean age was 27 years (range 16 to 53 years), with the majority being male (52.9%, *n* = 54) and having an American Society of Anaesthesiologists (ASA) grade of I (74.5%, *n* = 76). Of these 102 patients, the majority (*n* = 76, 74.5%) presented between the hours of 8 am and 8 pm. In the first 6 months after the opening of the ASAU, 106 were managed operatively for AA via the ASAU pathway (ASAU group) with a mean age of 26 years (age range 16 to 42 years). The majority in this group were also male (57.5%, *n* = 61) and with an ASA grade of I (72.6%, *n* = 77). Patients who presented after opening of the ASAU with suspected appendicitis but were not managed by the ASAU pathway due to the time of presentation being outside opening hours were excluded from the study.

### Time to be seen by the surgical team (Table 2)

**Table 2:** Comparison of outcomes between the ASAU group and the ED group

Key performance indicators	ASAU group (*n* = 106)	ED group (*n* = 102)	*p* value
Time to be seen by surgical team (min)	21 min	74 min	**<0.001**
Time to operating theatre (h)	12.5 h	21.3 h	**<0.001**
Utilisation of imaging (US or CT)	41.5% (*n* = 44)	30.3% (*n* = 31)	0.09
Consultant review on day of admission (%)	70.7% (*n* = 75)	41.2% (*n* = 42)	**<0.001**
Day-time operation (8 am–5 pm) (%)	78.6% (*n* = 83)	40.2% (*n* = 41)	**<0.001**
Evening-time operation (5 pm–12 am) (%)	18.9% (*n* = 20)	53.9% (*n* = 55)	**<0.001**
Nighttime operation (12am-8am) (%)	2.8% (*n* = 3)	5.8% (*n* = 6)	0.28
Consultant supervision in theatre (%)	76.4% (*n* = 81)	48% (*n* = 49)	**<0.001**
Conversion to open (%)	0.9% (*n* = 1)	1.9% (*n* = 2)	0.54
Perforated appendicitis (%)	5.7% (*n* = 6)	8.8% (*n* = 9)	0.38
Negative appendicectomy (%)	14.2% (*n* = 15)	18.6% (*n* = 19)	0.38
Inpatient length of stay of 1 night (%)	45.3% (*n* = 48)	31.4% (*n* = 32)	**0.03**
Inpatient length of stay of 2 nights (%)	49.1% (*n* = 52)	58.8% (*n* = 61)	0.12
Inpatient length of stay of more than 2 nights (%)	5.6% (*n* = 6)	8.8% (*n* = 9)	0.38
Mean length of stay (days)	2	2	1
Post-operative complications (%)	0.9% (*n* = 1)	3.9% (*n* = 4)	0.16
30-day readmission rate (%)	2.9% (*n* = 3)	0% (*n* = 0)	0.08

A *p* value less than 0.05 was deemed to be statistically significant

The time to be seen by the surgical team was defined as the time to be seen from referral by the ED triage nurse in the SAU group, and by the time to be seen from referral by the ED physician in the ED group. In the ED group, the mean time to be seen by a member of the on call surgical team from the time of referral was 74 min (range 18 to 196 min). In the ASAU group, the mean time to be seen was 21 min (range 5 to 54 min) (*p* < 0.001). All patients in the ASAU group were seen within 1 h of the time of referral.

### Utilisation of imaging

In the ED cohort, 31 patients (30.3%) had preoperative imaging to aid in the diagnosis of appendicitis. Of these, 17 (16.7%) underwent US and 14 (13.6%) underwent CT. This is compared to 44 patients (41.5%) in the ASAU group (*p* = 0.09), of which 17 (16%) underwent US and 27 (25.5%) underwent CT. The indications for imaging in most cases were diagnostic uncertainty for appendicitis, such as potential ovarian pathology in female patients that underwent US, or older patients with a broader potential that included possible malignancy and thus underwent CT.

### Time to theatre (Table 2)

In the ED group, the mean time from hospital presentation to arriving at the operating theatre was 21.3 h (1279 min), with the majority (65.7%, *n* = 67) of operations happening the day after admission. In the ASAU group, the mean time to theatre was 12.5 h (750 min), with a lower proportion (53%, *n* = 57) of operations having been deferred to the day after admission (*p* < 0.001).

### Time of day of surgery (Table 2)

The timing of surgery was classified as daytime (8 am to 5 pm), evening time (5 pm to midnight) and night time (midnight to 8 am). In the ED group, the majority had surgery in the evening (53.9%, *n* = 55), while the majority in the ASAU group (78.6%, *n* = 83) were operated on in daytime hours.

### Consultant supervision (Table 2)

Consultant supervision was defined as a consultant documented as being present in the operating theatre during surgery on the operating note. There was consultant supervision in 48% (*n* = 49) of cases in the ED group compared to 76.4% (*n* = 81) in the ASAU group (*p* = 0.001). A higher proportion of patients in the ASAU group were reviewed by a consultant on the day of admission (70.7%, *n* = 75) compared to the ED group (41.2%, *n* = 42) (*p* < 0.001). All patients in the ASAU group were seen by the consultant before proceeding to surgery, compared to 93.2% (*n* = 95) in the ED cohort (*p* = 0.02).

### Conversion to open surgery (Table 2)

In the ED group, two cases were converted from laparoscopic surgery to an open procedure via a gridiron incision, giving a conversion rate of 1.9%. The reason for conversion in both cases was due to technical difficulty and the presence of perforated appendicitis. One case was converted in the ASAU group for the same reason as in the ED group, with a conversion rate of 0.9% (*p* = 0.54). A consultant surgeon was present for all cases that were converted.

### Perforation rate (Table 2)

The presence of perforated appendicitis was confirmed by both the operation note and histopathology report in 9 patients (8.8%) in the ED group. In the ASAU group, 6 patients (5.7%) were found to have perforated appendicitis (*p* = 0.38).

### Inpatient length of stay (Table 2)

In the ASAU group, 45.3% (*n* = 48) spent one night in hospital, while in the ED group, only 31.4% (*n* = 32) spent one night in hospital (*p* = 0.03). In both groups, those that stayed five nights or more were kept in hospital for a prolonged course of intravenous antibiotics following surgery for perforated appendicitis. The mean length of stay was 2 nights in both cohorts of patients.

### Post-operative complications (Table 2)

The Clavien–Dindo classification system was used to define post-operative complications. In the ED group, 4 patients (3.9% complication rate) developed a post-operative complication. Two of these were wound infections requiring antibiotic treatment (Clavien–Dindo II complication). One patient developed a post-operative ileus which was managed conservatively (Clavien–Dindo I complication) and one patient developed a post-operative intra-abdominal collection which required radiological drainage. All of these complications were observed in patients who had perforated appendicitis. In the ASAU group, one patient (0.9% complication rate) represented with a wound infection treated with oral antibiotics. There were no reoperations or mortalities within 30 days of surgery recorded in either group.

### Thirty-day readmission rate

Three patients (2.9%) in the ED group were readmitted within the first 30 days following surgery. Two readmissions were for wound infections requiring intravenous antibiotics, one of which was a case that had been converted to open surgery. The other readmission in the ED group was a patient who represented with a post-operative collection requiring radiological drainage. No patients in the ASAU group were readmitted in the study period.

### Negative appendicectomy rate (Table 2)

In the ED group, the negative appendicectomy rate was 18.6% (*n* = 19) compared to 14.2% (*n* = 15) in the ASAU group (*p* = 0.38).

## Discussion

The management of EGS presents a major challenge for the healthcare system. As the workload of acute surgery continues to grow, new strategies are required to ensure the provision of timely and safe patient care [13, 14]. The introduction of acute medical assessment units (AMAUs) arose from the need to provide prompt consultant-led care to acute medical patients, while also serving to alleviate the workload and volume of patients in ED [15]. The benefits of this approach have been undeniable, resulting in a reduction of length of inpatient stay, readmission rates, mortality rates, trolley times, bed occupancy and waiting times to be seen by a doctor and for diagnostics [15]. It also came with significant cost savings and greater patient satisfaction [15]. The positive impact of the AMAU on both acute medical care and the ED workload highlights the need for similar models of care to be adapted in other disciplines. EGS in particular stands to benefit, where the volume of work continues to rise [16, 17]. The ASAU model of care has shown promising results in other healthcare systems, particularly in Australasia, but remains a relatively new venture in Ireland [6, 8–10]. Our study shows the positive impact that the implementation of the ASAU may have on one of the most common clinical problems in EGS.

### Key clinical outcomes

While the mean length of stay was equivalent in both cohorts, we observed that a statistically significant proportion of patients only required a hospital bed for a single night in the ASAU group compared to the ED group. The reasons for this are likely multifactorial. Given that the team covering the ASAU are not occupied with elective duties, they are free to attend to patients in a more timely manner, with all patients who presented via the ASAU pathway being seen within an hour of referral. This is in stark contrast to the ED group, where some patients waited more than three hours to be seen. With this more prompt review comes timelier decision making, the result of which meant patients in the ASAU cohort had a mean time to theatre that was nearly 10 h less than that of the ED patients. This allowed more patients to have their operation on the day of admission which facilitated earlier discharge. A greater consultant presence also likely contributed to this more efficient patient journey, with the majority of patients who presented via the ASAU having a same-day review by the most senior surgeon on call. Similar observations of improved patient flow have been made in ASAUs in both Australia and New Zealand, where emergency services that are separate from elective commitments have led to a more prompt time to theatre [12, 18, 19].

Shorter waiting times are of little value if they are not accompanied by improved clinical outcomes. Since our ASAU has become operational, we have observed a reduced negative appendicectomy rate, conversion rate, perforation rate and post-operative complication rate. It has been previously demonstrated that delays in management of appendicitis results in a higher perforation rate, which in turn leads to a higher rate of conversion to open surgery and more post-operative complications [20]. With this in mind, the quicker time to theatre in the ASAU group may improve clinical outcomes. The lower conversion-to-open rate in ASAU patients is likely a result of the greater consultant supervision that accompanies ASAU care. This is an important factor, with laparoscopic surgery being associated with less pain, a shorter length of stay and reduced post-operative wound infections [21]. Our study showed a lower negative appendicectomy rate in the ASAU cohort. With improved access to diagnostics and a higher degree of senior supervision comes lower negative appendicectomy rates, and our findings reflects this, resulting in less patients being put through the risk of a general anaesthetic and surgical procedure unnecessarily [22].

### Non-clinical outcomes

Some guidelines advocate for deferring emergency operations to daytime hours whenever possible, with one study suggesting that nighttime surgical mortality is double that of normal working hours due to surgeon fatigue, limited senior support and less availability of resources [23, 24].The ASAU may play an important role in facilitating surgery during daytime hours. In our study, significantly less patients in the ASAU group underwent surgery during night-time hours compared to the ED group. This is in line with studies performed in units abroad which also have demonstrated a reduction in potentially more dangerous out-of-hours operating [12, 23, 24].

The ASAU can provide an excellent teaching opportunity for trainee surgeons, creating an environment where they can assess a high volume of acute general surgical patients with senior supervision and separate to elective duties [6]. We demonstrated an increased consultant presence both in the assessment unit itself and in the operating theatre, which facilitates improved teaching and mentoring opportunities in the acute setting [8, 12]. A review of the operation notes revealed that, despite a greater consultant presence in theatre, the ASAU did not negatively impact the proportion of appendicectomies performed by trainees in our centre, but instead provided greater consultant presence to provide advice to the operating registrar, with the consultant documented as an assistant or not the primary operator in the vast majority of cases where they were present. The ASAU may also be a valuable resource for medical students, allowing them to meet acute surgical patients outside of the chaotic environment of the ED, where teaching opportunities can be challenging and unpredictable [8].

Making definitive conclusions about the impact of the ASAU on hospital savings is difficult. It is not hard to imagine that the implementation and opening of such a unit is highly costly, requiring a separate facility for patient assessment, as well as the cost of paying dedicated staff and running a dedicated emergency operating theatre. However, with an estimated cost of an inpatient hospital bed being €909 per night, the impact that the ASAU has on length of stay is an important consideration [25]. It is important to consider the potential financial benefits of a reduced length of stay and improved clinical outcomes offered by the ASAU in the context of the extra funding required to maintain such a unit. However, a systematic review and meta-analysis comparing ASAU with traditional ED care reported a cost saving of more than $1000 per patient managed via the ASAU [26]. Very few studies have discussed the financial impact of implementing an ASAU. A Department of Health report evaluating five consultant-led models of emergency general surgery in Victoria, Australia reported gains of $590′000 per annum, with cost savings resulting from a reduced inpatient length of stay, less use of ED resources and reduced night-time operating [27]. Considering the cost of opening the ASAU, the report concluded that the financial outcome was a break-even for the first year, with the potential for significant savings in the years beyond implementation [27]. We have demonstrated that our ASAU has resulted in a shorter length of stay, reduced night-time operating and has diverted patients away from a busy ED, which similarly provides significant potential for a positive impact on savings.

### The ASAU in Ireland

While the ASAU is a relatively new concept in the Irish healthcare system, the modest number of studies that focus on it have been favourable [6, 8–10]. However, these studies include a wide range of clinical conditions as part of a heterogenous case mix. A significant strength of our study is that it involved a homogenous cohort of patients with the same clinical condition, giving a clearer view of the impact of the shift from the ED model to the ASAU model on two similar patient cohorts. Our study is not without limitations. It was conducted retrospectively in a single centre. Our study also involved comparing an ED cohort that were admitted over a 24-h period to ASAU patients admitted during a 12-h period, which may have an impact on timeliness of access to definitive surgical management. Nonetheless, our findings are important, with the ASAU being relatively new to Ireland and thus very few studies assessing its value in the Irish context. To our knowledge, this is the first study in Ireland to demonstrate the impact of the ASAU on clinical outcomes in AA. The introduction of the ASAU model in AA leads to shorter time to theatre, shorter inpatient length of stay, greater consultant input, reduced conversion rate, reduced negative appendicectomy rate, less post-operative complications and improved teaching opportunities. Our findings support the role of the ASAU in improving the quality of care provided in EGS.

## Conclusions

We investigated the impact of introducing an ASAU on the outcomes of management of appendicitis. Statistically significant improvements were demonstrated across multiple KPIs. Patients waited less time to be seen, had a shorter time to theatre, a reduced length of inpatient stay and a lower post-operative complication rate. The conversion rate and negative appendicectomy rates were also lower. More operations were performed in safer daytime hours and a greater consultant presence was noted, allowing for greater teaching opportunities and senior support for trainee surgeons. To our knowledge, this is the first study in Ireland to specifically investigate the effect of the ASAU on outcomes in AA. Our study supports the role of the ASAU in improving the quality and efficiency of EGS.

## Data Availability

The data that supports the findings of this study is available from the corresponding author upon reasonable request. Data analysis was performed using the software package SPSS (SPSS Inc, Chicago, IL).

## Abbreviations

EGS: Emergency general surgery
ED: Emergency department
ASAU: Acute surgical assessment unit
NCPS: National Clinical Programme in Surgery
KPI: Key performance indicator
AA: Acute appendicitis
HIPE: Hospital inpatient enquiry
ASA: American society of anaesthesiologists
AMAU: Acute medical assessment unit
